# Only Extreme Fluctuations in Light Levels Reduce Lettuce Growth Under Sole Source Lighting

**DOI:** 10.3389/fpls.2021.619973

**Published:** 2021-01-28

**Authors:** Ruqayah Bhuiyan, Marc W. van Iersel

**Affiliations:** Horticultural Physiology Laboratory, Department of Horticulture, University of Georgia, Athens, GA, United States

**Keywords:** assimilation, chlorophyll, *Lactuca sativa*, light-emitting diodes, photosynthesis, photosynthetic photon flux density, variable electricity prices

## Abstract

The cost of providing lighting in greenhouses and plant factories can be high. In the case of variable electricity prices, providing most of the light when electricity prices are low can reduce costs. However, it is not clear how plants respond to the resulting fluctuating light levels. We hypothesized that plants that receive a constant photosynthetic photon flux density (PPFD) will produce more biomass than those grown under fluctuating light levels. To understand potential growth reductions caused by fluctuating light levels, we quantified the effects of fluctuating PPFD on the photosynthetic physiology, morphology, and growth of ‘Little Gem’ and ‘Green Salad Bowl’ lettuce. Plants were grown in a growth chamber with dimmable white LED bars, alternating between high and low PPFDs every 15 min. The PPFDs were ∼400/0, 360/40, 320/80, 280/120, 240/160, and 200/200 μmol⋅m^−2^⋅s^–1^, with a photoperiod of 16 h and a DLI of ∼11.5 mol⋅m^−2^⋅day^–1^ in all treatments. CO_2_ was ∼800 μmol⋅mol^–1^. Plants in the 400/0 μmol⋅m^−2^⋅s^–1^ treatment had ∼69% lower A_n_,_30_ (net assimilation averaged over 15 min at high and 15 min at low PPFD) than plants grown at a PPFD of 320/80 μmol⋅m^−2^⋅s^–1^ (or treatments with smaller PPFD fluctuations). The low A_n_,_30_ in the 400/0, and to a lesser extent the 360/40 μmol⋅m^−2^⋅s^–1^ treatment was caused by low net assimilation at 360 and 400 μmol⋅m^−2^⋅s^–1^. Plants grown at 400/0 μmol⋅m^−2^⋅s^–1^ also had fewer leaves and lower chlorophyll content compared to those in other treatments. The four treatments with the smallest PPFD fluctuations produced plants with similar numbers of leaves, chlorophyll content, specific leaf area (SLA), dry mass, and leaf area. Chlorophyll content, A_n_,_30_, and dry mass were positively correlated with each other. Our results show that lettuce tolerates a wide range of fluctuating PPFD without negative effects on growth and development. However, when fluctuations in PPFD are extreme (400/0 or 360/40 μmol⋅m^−2^⋅s^–1^), chlorophyll levels and A_n_,_30_ are low, which can explain the low poor growth in these treatments. The ability of lettuce to tolerate a wide range of fluctuating light levels suggests that PPFD can be adjusted in response to variable electricity pricing.

## Introduction

Increased year-round demand for fresh fruits and vegetables has increased the need for productive and profitable controlled environment growing operations, such as greenhouses and plant factories. Among the most popular crops for controlled environment agriculture are various leafy greens, including lettuce (Agrylist, 2017). Because of large day-to-day and seasonal fluctuations in the daily light integral (DLI) from sunlight (Albright et al., 2000), consistent, year-round greenhouse production of lettuce may require supplemental lighting from Fall through Spring. This is especially important at higher latitudes, where seasonal fluctuations in DLI from sun are greatest (Faust and Logan, 2018). However, the light environment in greenhouses is often poorly controlled (van Iersel and Gianino, 2017) and the variable light environment makes greenhouse production less predictable. The capital and operating costs of supplemental lighting are high (Albright et al., 2000). Lighting accounts for up to 30% of total operating costs in greenhouses (van Iersel and Gianino, 2017) and 40–50% in plant factories, either to provide the light or to remove the heat generated by the light fixtures (Watanabe, 2011; Zeidler and Schubert, 2014). Reducing the cost of lighting in controlled environment agriculture can reduce operating costs and increase profitability. One potential approach to decrease the cost of supplemental lighting is the use of photovoltaic greenhouses, where part of the greenhouse roof is covered with solar panels (Emmott et al., 2015; Cossu et al., 2017). However, the resulting shading of the greenhouse crop can reduce yields (Cossu et al., 2020). In addition, photovoltaic panels generate most electricity when there is ample sunlight, so there is a disconnect between the availability of electricity from photovoltaic panels and the need for supplemental lighting. Although the power generated by photovoltaic panels can be stored in batteries, this is expensive.

One obvious option for reducing electricity costs is to take advantage of variable electricity prices. The Light and Shade System Implementation (LASSI) algorithm can account for variable electricity prices and was shown to reduce electricity costs of greenhouse production by 8–37% as compared to threshold lighting control, where lights are controlled based on PPFD readings. The magnitude of the cost savings depended on location and which threshold control algorithm LASSI was compared to Harbick et al. (2016). Sørensen et al. (2016) used a multi-objective evolutionary algorithm in their DynaGrow control system to optimize greenhouse temperature, CO_2_, and supplemental lighting, based on the greenhouse environment, electricity price forecasts, and weather forecasts. DynaGrow successfully reduced energy use and cost, while resulting in similar quality plants as a standard lighting control approach. Based on this prior work, accounting for energy prices in control algorithms for supplemental light can reduce energy costs. However, Kjaer et al. (2011) showed that an irregular greenhouse light environment resulted in poor flowering of *Campanula*, which could be prevented by assuring that the photoperiod was the same each day.

How fluctuating light levels affect photosynthetic physiology in controlled environments is not clear. Leaves in outdoor canopies experience changes in PPFD in the form of sunflecks, lasting anywhere from a few seconds to a few minutes, and shadeflecks, due to cloud cover, which can last hours (Knapp and Smith, 1987). The occurrence of sunflecks is dependent on movement of the sun and/or leaves higher in the canopy. Understory plants have adapted to the occurrence of sunflecks and have developed photosynthetic machinery to facilitate efficient use of this high PPFD (Chazdon and Pearcy, 1991). When plants are exposed to high light after periods of low light or darkness, it can take 10–40 min for leaves to acclimate and reach steady state photosynthesis, and is dependent on the duration and timing of those sunflecks (Chazdon and Pearcy, 1986, 1991). Vice versa (Kromdijk et al., 2016) showed that downregulation of photoprotective mechanisms as sunlit leaves are suddenly shaded can be slow, reducing photosynthesis of those shaded leaves. Upregulating the expression of genes encoding violaxanthin de-epoxidase, zeaxanthin epoxidase, and PSII subunit S allowed plants to respond more quickly to sudden reductions in sunlight increased dry matter production of tobacco (*Nicotiana tabacum*) by 15%.

Vialet-Chabrand et al. (2017) compared the photosynthetic physiology and growth of *Arabidopsis thaliana* under four different lighting treatments, constant high or low PPFD during the entire photoperiod vs. natural fluctuations in PPFD, resulting in the same DLI. Plants grown with a greater DLI had a higher light-saturated rate of photosynthesis, but whether that DLI was provided with constant or fluctuating PPFD had little impact on the photosynthetic physiology. However, fluctuating PPFD resulted in thinner leaves, decreased leaf area, and shoot biomass, and increased specific leaf area (SLA), as compared to constant PPFD with the same DLI. This reduction in growth under fluctuating PPFD was at least partly explained by a greater daily net carbon gain (photosynthesis minus respiration) under constant as compared to fluctuating PPFD (Vialet-Chabrand et al., 2017). These differences in daily net carbon gain are likely caused by multiple factors. First, under fluctuating PPFD conditions, plants are required to constantly acclimate to a changing light environment, which can reduce photosynthetic efficiency and growth (Kromdijk et al., 2016). Secondly, because of the asymptotic shape of photosynthesis-light response curves (Vialet-Chabrand et al., 2017), the total photosynthesis over the course of a day, given a specific DLI, is achieved under constant PPFD conditions (Sims and Pearcy, 1993). Likewise, the daily electron transport rate, the photosynthetic process most directly impacted by light, with a specific DLI increases as PPFD fluctuations decrease (Weaver and van Iersel, 2019).

Our objective was to quantify the photosynthesis and growth of lettuce in response to fluctuating PPFD levels. We hypothesized that plant biomass would decrease as the magnitude of PPFD fluctuations increased, because of the effect of such fluctuations on photosynthesis and carbon gain. By quantifying the effects of fluctuating PPFD on plant physiological parameters and crop growth, we aimed to determine whether it is possible to take advantage of variable electricity prices to provide light to controlled environment agriculture crops.

## Materials and Methods

### Growing Conditions

The study was conducted in a 54 m^3^ walk-in growth chamber. The chamber contained three racks with three shelves each. Each shelf was divided into two 0.74 m^2^ growing areas. Each growing area was outfitted with two dimmable LED bars (SPYDRx with Physiospec indoor spectrum, Fluence Bioengineering, Austin, TX, United States). Environmental conditions were monitored with a temperature/humidity probe (HMP50, Vaisala, Helsinki, Finland) and a CO_2_ sensor (GMC20, Vaisala, Vantaa, Finland) connected to a datalogger (CR6, Campbell Scientific, Logan, UT, United States), which calculated the vapor pressure deficit (VPD) from the temperature and relative humidity measurements. The datalogger controlled CO_2_ levels by opening a valve connected to a compressed CO_2_ cylinder for 0.1 s, whenever the measured CO_2_ dropped below 800 μmol⋅mol^–1^. CO_2_ enrichment was used because it can make supplemental lighting more economical by increasing photosynthesis and growth more than supplemental lighting by itself (Both et al., 1997; Ferentinos et al., 2000). Excess water vapor was removed using a dehumidifier (FAD704DWD13, Electrolux, Charlotte, NC, United States). The temperature was 19.7 ± 0.8°C, CO_2_ concentration was 797 ± 47 μmol⋅mol^–1^, and the VPD was 0.99 ± 0.17 kPa (mean ± *SD*).

### Plant Material

Lettuce ‘Green Salad Bowl’ and ‘Little Gem’ were seeded into 10-cm square pots filled with peat-perlite substrate (Fafard 2P; Sun Gro Horticulture, Agawam, MA, United States). Seedlings were thinned to one plant per pot at 6 days after seeding. Plants were sub-irrigated as needed using a water-soluble fertilizer solution with a nitrogen concentration of 100 mg⋅L^–1^ (Peters Excel 15-5-15 CalMag Special, ICL, Summerville, SC, United States). The experimental unit was a group of 15 plants of one cultivar, with three replications, and six treatments (PPFD fluctuations). The plants were grown over a 6 weeks period.

### Treatments

Plants were grown under six different fluctuating lighting treatments with the photosynthetic photon flux density (PPFD) switching from high to low PPFD every 15 min throughout the photoperiod. The PPFDs in the different treatments were approximately 400/0, 360/40, 320/80, 280/120, 240/160, and 200/200 μmol⋅m^−2^⋅s^–1^, with a photoperiod of 16 h. The DLI in all treatments was ∼11.5 mol⋅m^−2^⋅day^–1^. The actual PPFD was not exactly equal to the target PPFD and measured using a spectroradiometer (SS-110, Apogee, Logan, UT) (Table 1). Measurements were taken at 9 cm height from the ebb-and-flow tray, 3 cm above the soil line at the center of each 15-unit tray.

**TABLE 1:** Target PPFDs (mean ± *SD*; *n* = 3) and actual measured PPFDs for each fluctuating lighting treatment.

Target high and low PPFD (μmol⋅m^−2^⋅s^–1^)	High PPFD (μmol⋅m^−2^⋅s^–1^)	Low PPFD (μmol⋅m^−2^⋅s^–1^)
200/200	211 ± 5	211 ± 5
240/160	249 ± 4	167 ± 4
280/120	283 ± 5	123 ± 3
320/80	341 ± 8	86 ± 2
360/40	367 ± 19	41 ± 2
400/0	420 ± 16	0.2 ± 0.1

*Data was collected at canopy level.*

### Data Collection and Analysis

Canopy images of trays with 15 plants were taken weekly after seedling emergence [16, 23, 30, and 37 days after planting (DAP)]. We used a monochrome camera (CM3-U3-31S4M-CS, Flir, Wilsonville, OR, United States) outfitted with a 680 nm long-pass filter (Midwest Optics, Palatine, IL, United States) mounted inside a light-proof grow tent. Plants were illuminated with a blue LED (225 ultrathin grow light, Yescom United States, City of Industry, CA, United States). The camera took images of the fluorescence emitted by the leaves, excited by the blue light, resulting in grayscale images, with the canopy light and the background dark. The projected canopy size for each tray of plants was determined using threshold separation in ImageJ (Narayanan et al., 2019).

Gas exchange data was collected on one ‘Green Salad Bowl’ plant per experimental unit at 35–37 DAP to determine the photosynthesis of plants within each treatment using a portable leaf gas exchange system (CIRAS-3, PP Systems, Inc., Amesbury, MA). The youngest fully expanded leaf was used for these measurements. The leaf gas exchange system was programmed to run for 45 min; 15 min of low PPFD (as an acclimation period), followed by 15 min of high and 15 min of low PPFD. Built in white LEDs were programmed to set the target PPFDs in the leaf cuvette. Cuvette temperature, CO_2_ concentration, and VPD were similar to conditions in the growth chamber. The net assimilation data for each 15 min period were averaged (A_n_,_15_), as were the data from the 30 min period, which included 15 min of both high and low PPFD (A_n_,_30_). Stomatal conductance was measured as well.

‘Green Salad Bowl’ was harvested at 40 DAP and ‘Little Gem’ was harvested at 43 DAP. The chlorophyll content index (CCI) (Opti-Sciences, CCM-200plus, Hudson, NH), number of leaves, length and width of the longest leaf, total leaf area, and shoot dry weight were measured on the three plants in the center of each tray. SLA was calculated as leaf area/shoot dry weight. Dry mass measurements were collected from the 12 remaining border plants for calculating total dry mass.

### Experimental Design and Statistical Analysis

The study was set up as a randomized complete block with three replications and a split-plot (cultivar). Data was analyzed using both linear and non-linear regression (SigmaPlot 11, Systat Software, Inc., San Jose, CA).

## Results

### Crop Growth and Morphology

**FIGURE 1:**
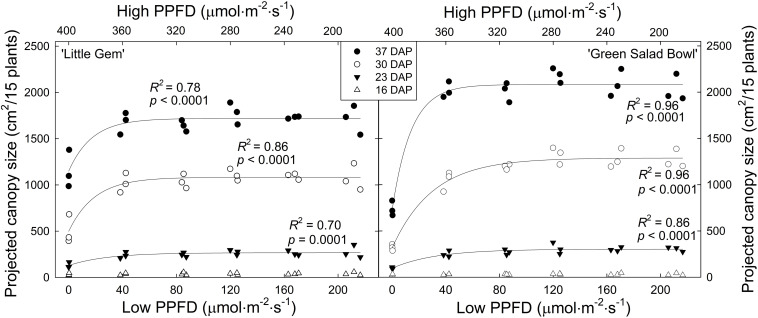
Projected canopy size of ‘Little Gem’ and ‘Green Salad Bowl’ lettuce (*Lactuca sativa*) at 16, 23, 30, and 37 days after planting (DAP), measured on experimental units consisting of 15 plants. Plants were grown under fluctuating photosynthetic photon flux density (PPFD), with PPFD changing every 15 min between high and low intensities (∼400/0, 360/40, 320/80, 280/120, 240/160, and 200/200 μmol⋅m^−2^⋅s^–1^). Identical symbols represent the three replications of each treatment.

Projected canopy size at 16 DAP was low and not affected by PPFD fluctuations for either cultivar. At all subsequent times, PPFD fluctuations did affect projected canopy size, with 400/0 μmol⋅m^−2^⋅s^–1^ fluctuations resulting in the smallest canopy size in both cultivars. In ‘Green Salad Bowl,’ the 360/40 μmol⋅m^−2^⋅s^–1^ treatment resulted in slightly lower projected canopy size than treatments with smaller PPFD fluctuations at 23 and 30 DAP, but no longer at 37 DAP (Figure 1).

Projected canopy size of ‘Green Salad Bowl’ was more sensitive to PPFD fluctuations than that of ‘Little Gem’; at 37 DAP, projected canopy size of ‘Little Gem’ was 32% lower with 400/0 μmol⋅m^−2^⋅s^–1^ fluctuations than in the other treatments, while for ‘Green Salad Bowl,’ this reduction was 64%. In treatments with PPFD fluctuations of 360/40 μmol⋅m^−2^⋅s^–1^ or less, ‘Little Gem’ had a ∼12.5% smaller projected canopy than ‘Green Salad Bowl’ at 37 DAP (Figure 1). This is consistent with the growth habits of these two cultivars; ‘Green Salad Bowl’ is a loose-leaf lettuce, while ‘Little Gem’ forms a small head.

**FIGURE 2:**
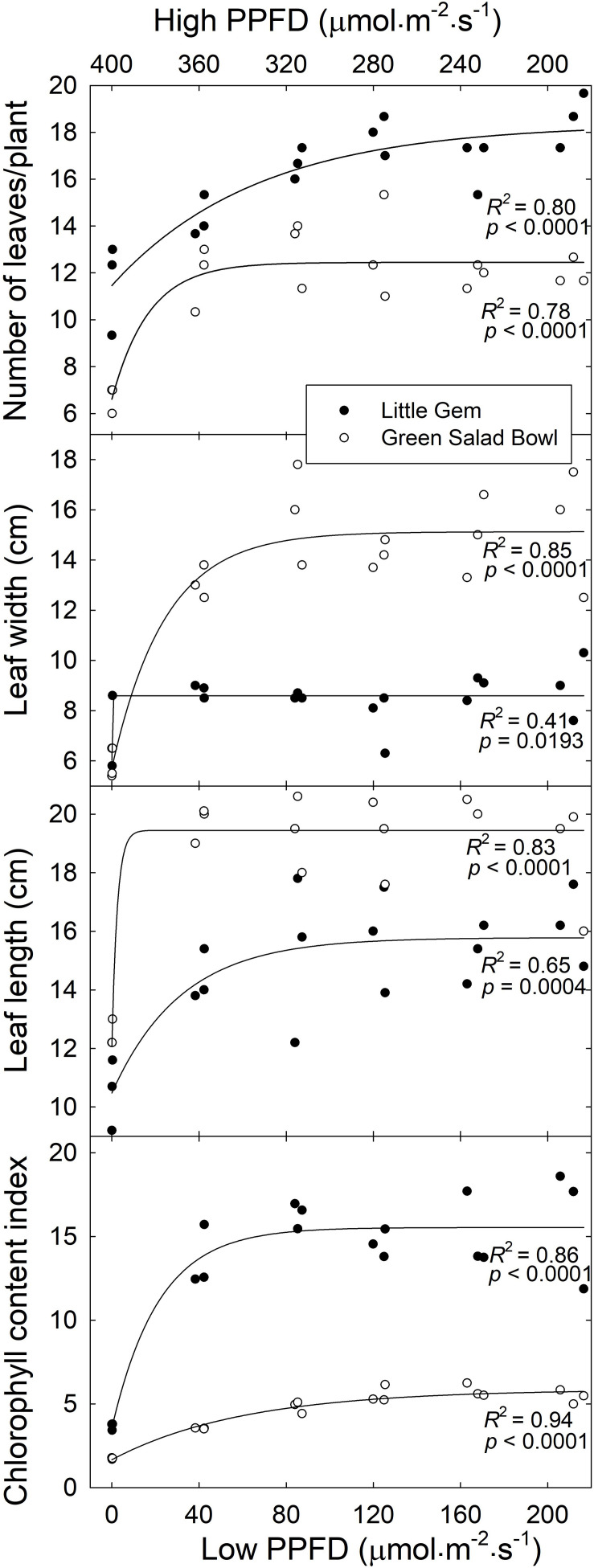
Number of leaves per plant, leaf width, leaf length, and chlorophyll content index as a function of treatment (*x*-axis indicates lower *PPFD*). Symbols (three replications per treatment) represent cultivars ‘Green Salad Bowl’ (open symbols) and ‘Little Gem’ (closed symbols) of lettuce (*Lactuca sativa*). Measurements are from the three center plants from each tray. Plants were grown under fluctuating photosynthetic photon flux density (*PPFD*), with *PPFD* changing every 15 min between high and low intensities (∼400/0, 360/40, 320/80, 280/120, 240/160, and 200/200 μmol⋅m^−2^⋅s^–1^).

In both cultivars, there was an asymptotic increase in leaf number, length, width, and chlorophyll content index. ‘Green Salad Bowl’ plants averaged 6.7 leaves in the 400/0 μmol⋅m^−2^⋅s^–1^ treatment, compared to 12.3 leaves in the other treatments (Figure 2). For ‘Little Gem,’ plants in the 400/0 μmol⋅m^−2^⋅s^–1^ treatment averaged 11.6 leaves, increasing to 14.3 leaves in the 360/40 μmol⋅m^−2^⋅s^–1^ treatment and 17.4 leaves in the other treatments (Figure 2). Leaf length of ‘Green Salad Bowl’ averaged 12.5 cm in the 400/0 μmol⋅m^−2^⋅s^–1^ treatment, compared to 19.4 cm in the other treatments. ‘Little Gem’ plants in the 400/0 μmol⋅m^−2^⋅s^–1^ treatment averaged a leaf length of 10.5 cm, increasing to 14.4 cm in the 360/40 μmol⋅m^−2^⋅s^–1^ and 16.6 cm for the other treatments (Figure 2). Leaf width for ‘Green Salad Bowl’ averaged 5.8 cm in the 400/0 μmol⋅m^−2^⋅s^–1^ treatment, increasing to 13.1 cm in the 360/40 μmol⋅m^−2^⋅s^–1^ treatments and 15.1 cm in all other treatments. For ‘Little Gem,’ the 400/0 μmol⋅m^−2^⋅s^–1^ treatment resulted in a leaf width of 7.0 cm, increasing to 8.6 cm in the other treatments (Figure 2).

‘Green Salad Bowl’ had an ∼67% lower chlorophyll content index than ‘Little Gem.’ Plants grown under the 400/0 μmol⋅m^−2^⋅s^–1^ treatment had an ∼65 and ∼75% lower chlorophyll content index as compared to the other treatments in ‘Green Salad Bowl’ and ‘Little Gem,’ respectively. ‘Green Salad Bowl’ had larger but fewer leaves than ‘Little Gem’ and the number of leaves increased more gradually, from ∼12 to 18, for ‘Little Gem than for Green Salad Bowl’ (∼7–12 leaves), as PPFD fluctuations decreased (Figure 2 and Supplementary Figure S1).

**FIGURE 3:**
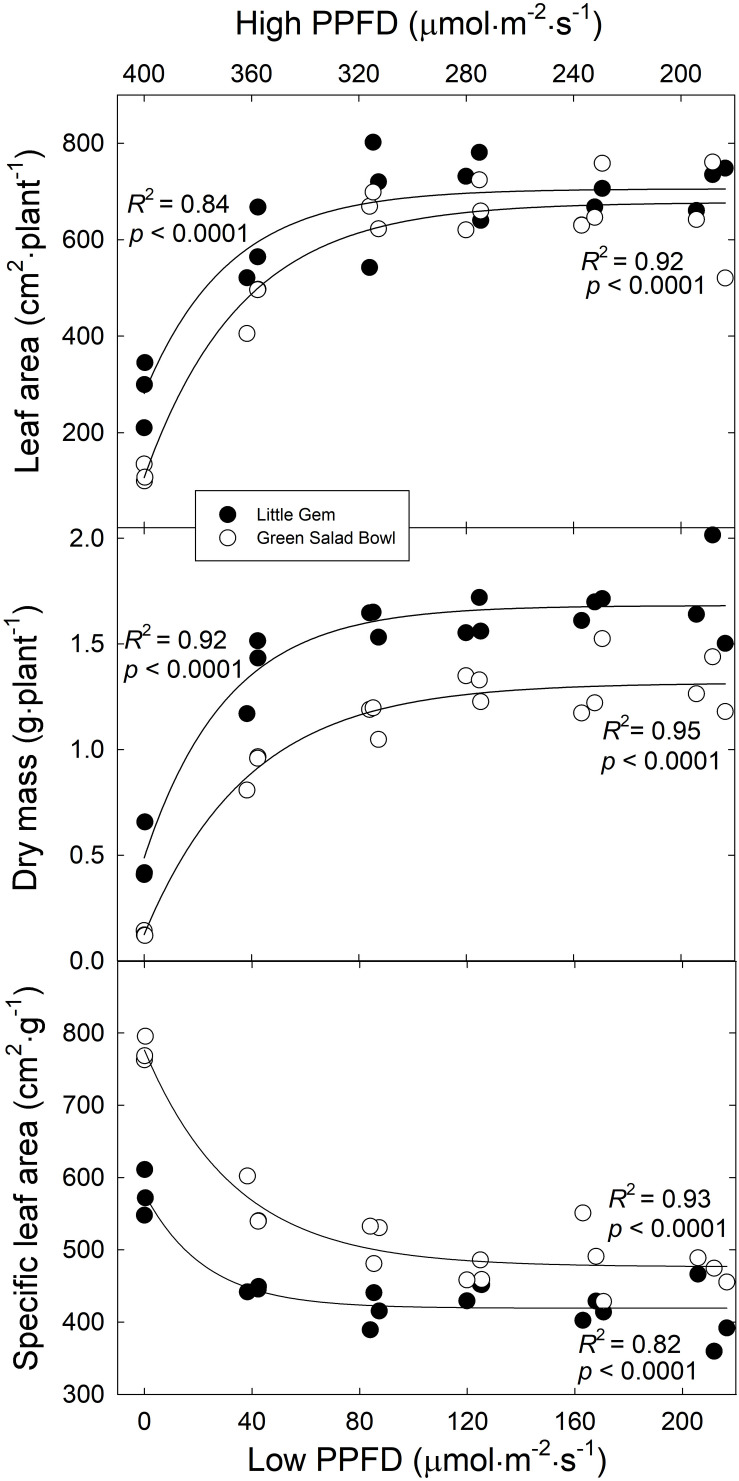
Leaf area per plant, dry mass per plant, and specific leaf area as a function of the treatments (*x*-axis indicates lower PPFD). Symbols (three replications per treatment) represent cultivars ‘Green Salad Bowl’ (open symbols) and ‘Little Gem’ (closed symbols) of lettuce (*Lactuca sativa*). Measurements from the three center plants from each tray. Plants were grown under fluctuating photosynthetic photon flux density (PPFD), with PPFD changing every 15 min between high and low intensities (∼00/0, 360/40, 320/80, 280/120, 240/160, and 200/200 μmol⋅m^−2^⋅s^–1^).

On average, ‘Little Gem’ had an ∼8% larger leaf area than ‘Green Salad Bowl’ (Figure 3), which contrasts with the substantially larger projected canopy size of ‘Green Salad Bowl.’ This is likely related to the compact and head-forming ‘Little Gem’ having smaller but more leaves (Figure 2), which overlap each other more than the leaves of the loose-leaf ‘Green Salad Bowl’ lettuce.

Leaf area and total dry mass of both cultivars increased asymptotically as the lower PPFD increased from 0 to 200 μmol⋅m^−2^⋅s^–1^. ‘Green Salad Bowl’ plants in the 400/0 μmol⋅m^−2^⋅s^–1^ and 360/40 μmol⋅m^−2^⋅s^–1^ treatments, had a ∼90 and ∼28% lower dry mass and an ∼83 and ∼30% lower leaf area compared to the other treatments (Figure 3). ‘Little Gem’ plants in the 400/0 μmol⋅m^−2^⋅s^–1^ and 360/40 μmol⋅m^−2^⋅s^–1^ treatments, had a ∼70 and ∼22% lower dry mass and an ∼59 and ∼16% lower leaf area compared to the other treatments. ‘Green Salad Bowl’ had a ∼12% lower dry mass and ∼28% lower leaf area than ‘Little Gem’ (Figure 3).

Specific leaf area decreased exponentially as the lower PPFD increased from 0 to 200 μmol⋅m^−2^⋅s^–1^, suggesting thinner leaves with large PPFD fluctuations in both lettuce cultivars. The SLA of ‘Green Salad Bowl’ plants in the 360/40 μmol⋅m^−2^⋅s^–1^ treatment was ∼28%, and in those with smaller PPFD fluctuations ∼37%, lower than in the 400/0 μmol⋅m^−2^⋅s^–1^ treatment (Figure 3). ‘Little Gem’ SLA in the 360/40 μmol⋅m^−2^⋅s^–1^ treatment was ∼23%, and in the other treatments ∼27% lower than in the 400/0 μmol⋅m^−2^⋅s^–1^ treatment. ‘Little Gem’ had a ∼18% lower SLA than ‘Green Salad Bowl’ (Figure 3).

### Leaf Assimilation Rates

**FIGURE 4:**
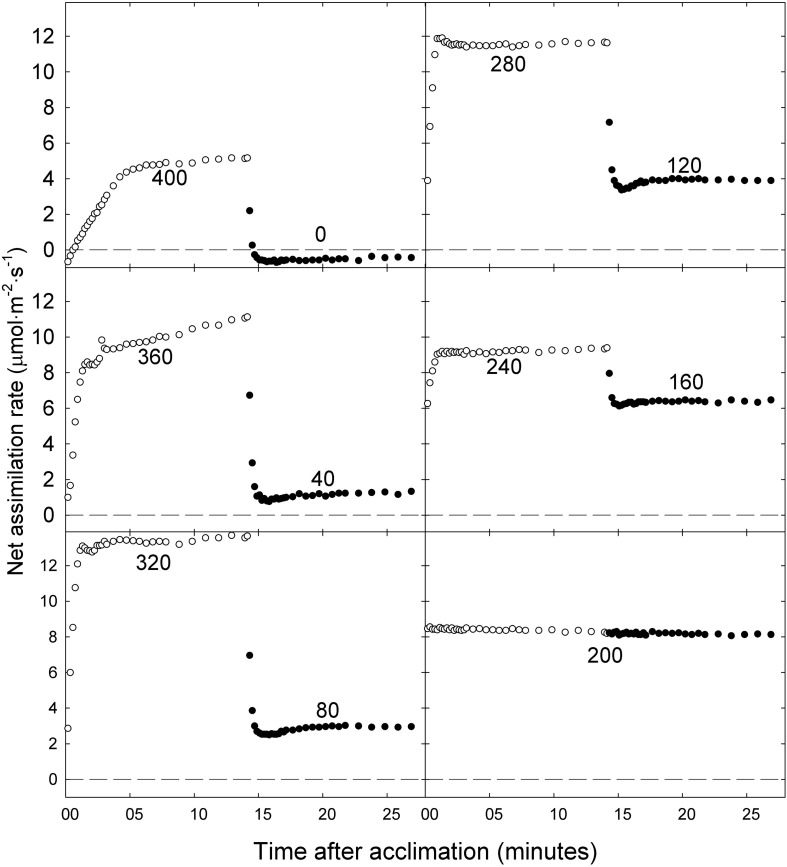
Net photosynthetic rate of ‘Green Salad Bowl’ lettuce (*Lactuca sativa*) during a 15 min high photosynthetic photon flux density (PPFD) period followed by a 15 min low PPFD period (400/0, 360/40, 320/80, 280/120, 240/160, and 200/200 μmol⋅m^−2^⋅s^–1^). Open circles represent high PPFD, and closed circles low PPFD. Values in each graph indicate the PPFD.

Net assimilation rates of ‘Green Salad Bowl’ lettuce in most treatments increased rapidly as the PPFD was changed from low to high. However, plants in the 400/0 μmol⋅m^−2^⋅s^–1^ treatment, and to a lesser extent the 360/40 μmol⋅m^−2^⋅s^–1^ treatment, showed a more gradual initial increase in A_n_ (for about 5 min) following exposure to high PPFD. Net assimilation did not reach a steady state during the 15 min at high PPFD in the 400/0 and 360/40 μmol⋅m^−2^⋅s^–1^ treatments, but instead kept increasing slowly (Figure 4). This suggests that the plants may have been trying to acclimate to the high PPFD but were not able to fully do so before the PPFD was lowered again. In all other treatments, stable A_n_ was reached within 2 min at high PPFD. After switching from high to low PPFD, A_n_ stabilized quickly in all treatments. The 200/200 μmol⋅m^−2^⋅s^–1^ treatment resulted in consistent A_n_ over the 30 min period, ranging between 8.1 and 8.6 μmol⋅m^−2^⋅s^–1^ (Figure 4).

**FIGURE 5:**
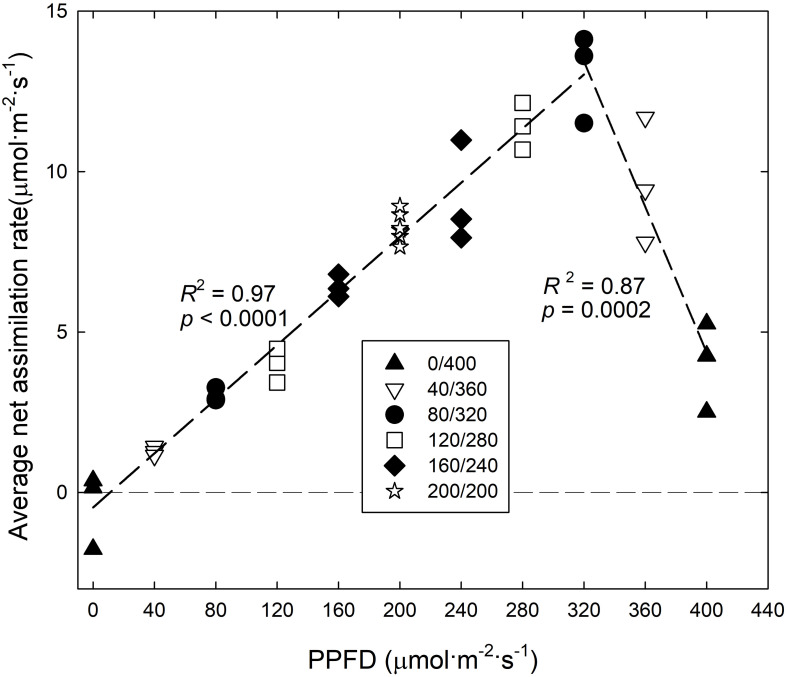
Average net assimilation rate over 15 min (A_n, 15_) of ‘Green Salad Bowl’ lettuce (*Lactuca sativa*) as a function of photosynthetic photon flux density (PPFD). Photosynthesis was measured for 15 min under high PPFD, followed by 15 min under low PPFD (see Figure 2). Plants were grown under fluctuating PPFD, changing every 15 min between high and low PPFD (∼400/0, 360/40, 320/80, 280/120, 240/160, and 200/200 μmol⋅m^−2^⋅s^–1^).

The A_n_,_15_ of ‘Green Salad Bowl’ lettuce increased linearly, from ∼-1 to 14 μmol⋅m^−2^⋅s^–1^, as PPFD increased from 0 to 320 μmol⋅m^−2^⋅s^–1^ and decreased rapidly at even higher PPFDs. At 400 μmol⋅m^−2^⋅s^–1^, A_n_,_15_ averaged only ∼4 μmol⋅m^−2^⋅s^–1^, ∼9.1 μmol⋅m^−2^⋅s^–1^ lower than at a PPFD of 320 μmol⋅m^−2^⋅s^–1^ (Figure 5), indicating that the extreme PPFD fluctuations in the 400/0 μmol⋅m^−2^⋅s^–1^ treatment seriously impaired the photosynthetic physiology.

**FIGURE 6:**
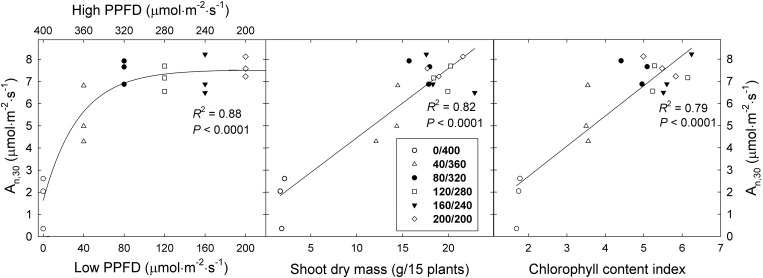
Average net assimilation rate over 30 min (A_n, 30_) of ‘Green Salad Bowl’ lettuce (*Lactuca sativa*) as a function of fluctuating photosynthetic photon flux density (PPFD), total dry mass, and chlorophyll content index. Symbols represent data from each lighting treatment (three replications per treatment). Plants were grown under fluctuating PPFD, changing every 15 min between high and low PPFD (∼400/0, 360/40, 320/80, 280/120, 240/160, and 200/200 μmol⋅m^−2^⋅s^–1^).

The A_n_,_30_ of ‘Green Salad Bowl’ lettuce increased asymptotically as the lower PPFD increased from 0 to 200 μmol⋅m^−2^⋅s^–1^ (and the high PPFD decreased from 400 to 200 μmol⋅m^−2^⋅s^–1^), with little or no difference among the 320/80, 280/120, 240/160, and 200/200 μmol⋅m^−2^⋅s^–1^ treatments (Figure 6). The linear relationship between A_n_,_15_ at PPFDs from 0 to 320 μmol⋅m^−2^⋅s^–1^ (Figure 5) explains the lack of differences A_n_,_30_ among these four treatments (Figure 6).

The A_n_,_30_ in the 360/40 and 400/0 treatments was ∼27 and 69% lower compared to the other treatments with smaller PPFD fluctuations. The rapid decrease in A_n_,_15_ at a PPFD above of 320 μmol⋅m^−2^⋅s^–1^ (Figure 5) explains the low A_n_,_30_ in the two treatments with the greatest PPFD fluctuations.

The A_n_,_30_ data follow the same trends as the dry mass and leaf area data (Figure 3). There was a strong positive correlation between the A_n_,_30_ and shoot dry mass of ‘Green Salad Bowl’ lettuce, largely due to the low A_n_,_30_ and dry mass in the 400/0 μmol⋅m^−2^⋅s^–1^ treatment (Figure 6). Since A_n_ underlies dry mass production, this correlation is not surprising. The A_n_,_30_ of ‘Green Salad Bowl’ lettuce also was positively correlated with the leaf chlorophyll content index (Figure 6), suggesting that the low A_n_ and dry mass of plants grown under a PPFD of 400/0 μmol⋅m^−2^⋅s^–1^ were at least partly due to the low chlorophyll levels in the leaves of these plants.

**FIGURE 7:**
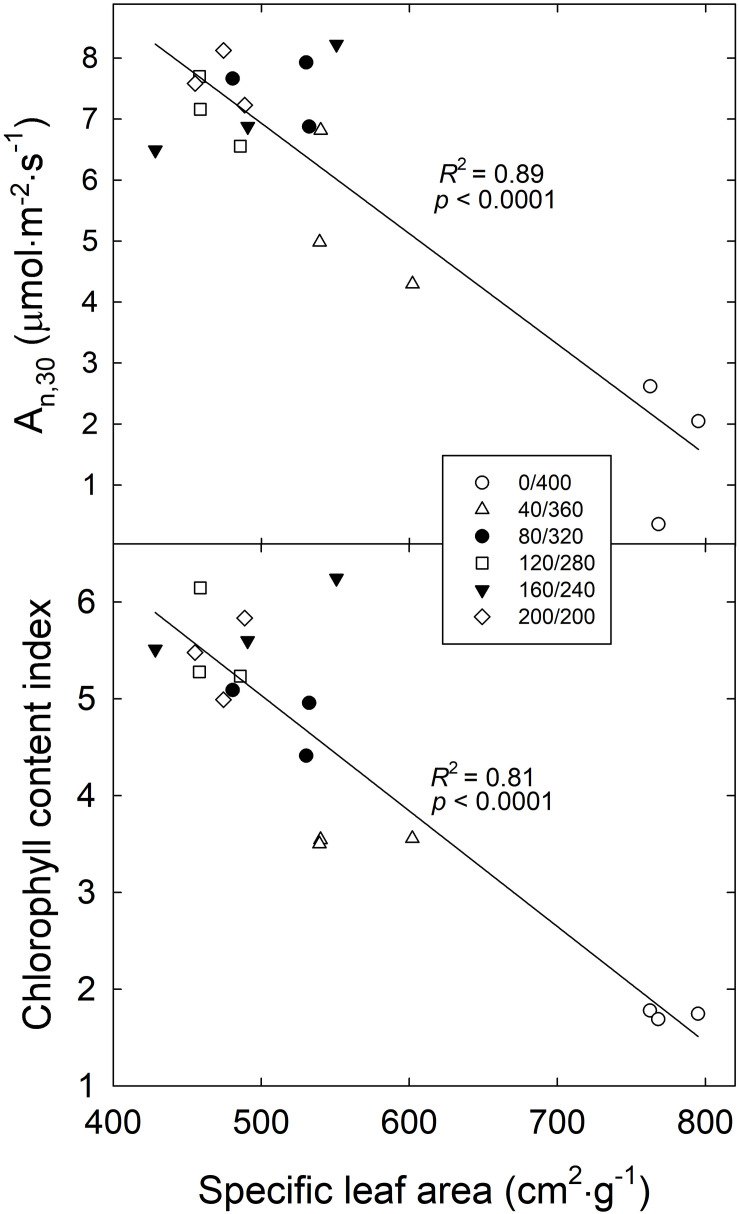
Average net photosynthesis over a 30 min period (A_n,30_) and chlorophyll content index of ‘Green Salad Bowl’ lettuce (*Lactuca sativa*) as a function of specific leaf area. Symbols represent each treatment, with three replications per treatment. Plants were grown and measured under fluctuating PPFD, changing every 15 min between high and low PPFD (∼ 400/0, 360/40, 320/80, 280/120, 240/160, and 200/200 μmol⋅m^−2^⋅s^–1^).

SLA of ‘Green Salad Bowl’ lettuce was negatively correlated with both A_n_,_30_ and CCI (Figure 7). High SLA suggests thinner leaves with fewer and/or smaller mesophyll cells, where most of the carbon assimilation occurs. As the SLA decreased from ∼780 cm^2^⋅g^–1^ (in the 400/0 μmol⋅m^−2^⋅s^–1^ treatment) to 460 cm^2^⋅g^–1^, A_n_,_30_ increased from 1.9 to 7.6 μmol⋅m^−2^⋅s^–1^ and CCI increased from 1.7 to 5.4 (Figure 7).

## Discussion

### The Importance of Canopy Size

Projected canopy size (PCS) is a good indicator of the amount of light a canopy intercepts (Klassen et al., 2004) and of morphological changes in response to environmental conditions, in this case fluctuations in PPFD. When taken over a growing period, it provides information on growth rates from seed to maturity. In the 400/0 μmol⋅m^−2^⋅s^–1^ treatment for both cultivars, the plants had a lower PCS than those in other treatments throughout the growing period from 23 DAP until the end of the study (Figure 1). A lower projected canopy size reduces the amount of incident light, canopy photosynthesis, and growth (Klassen et al., 2004). Projected canopy sizes in all other treatments were similar, indicating that lettuce canopy development tolerates wide fluctuations in PPFD.

**FIGURE 8:**
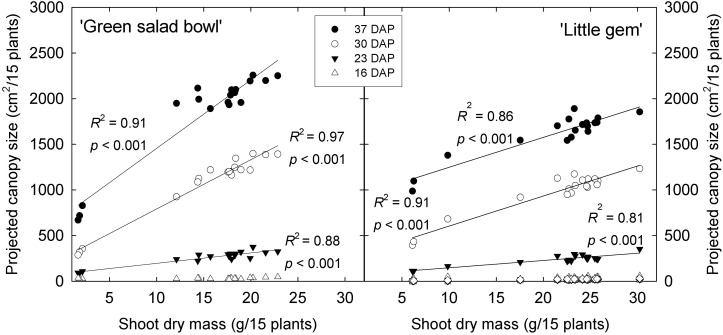
Projected canopy size of ‘Green Salad Bowl’ and ‘Little Gem’ lettuce (*Lactuca sativa*) at 16, 23, 30, and 37 days after planting (DAP) vs. shoot dry mass of 15 plants. Symbols represent DAP. Plants were grown under fluctuating *PPFD*, with *PPFD* changing every 15 min between high and low intensities (∼400/0, 360/40, 320/80, 280/120, 240/160, and 200/200 μmol⋅m^−2^⋅s^–1^).

The PCS of ‘Green Salad Bowl’ was more sensitive to large PPFD fluctuations than that of ‘Little Gem.’ At 30 and 37 DAP, ‘Green Salad Bowl’ had a larger PCS than ‘Little Gem’ in treatments with relatively small PPFD fluctuations (200/200 to 320/80 μmol⋅m^−2^⋅s^–1^), while ‘Green Salad Bowl’ had a smaller PCS in the 400/0 μmol⋅m^−2^⋅s^–1^. This indicates that genetic factors play a role in determining both PCS, as well as cultivar responses to fluctuating PPFD. ‘Green Salad Bowl’ produces larger leaves than ‘Little Gem,’ a small head-forming lettuce (Figure 2). The importance of PCS in determining crop growth is evident from the positive correlation between PCS at 23, 30, and 37 DAP and final dry mass (Figure 8). Our results suggest that measurements of PCS during the growing cycle can provide an early indication of final dry mass production in response to different lighting treatments. Similar correlations between PCS and final dry mass were reported by Elkins and van Iersel (2020) in response to different PPFD and photoperiod treatments, all with the same DLI. Differences in growth among lettuce cultivars are also strongly correlated with differences in canopy size early in the growing cycle (Kim and van Iersel, 2019).

The effects of fluctuating light levels on PCS were consistent with effects on leaf number, length, width, and total leaf area in both cultivars (Figures 2, 3). These treatment effects tended to be larger in ‘Green Salad Bowl’ than in ‘Little Gem.’ The reductions in these morphological parameters in response to the 400/0 μmol⋅m^−2^⋅s^–1^, and to a lesser extent in the 360/40 μmol⋅m^−2^⋅s^–1^ treatments, may be the result the low A_n_,_30_ (Figure 6) and the resulting limited carbohydrate supply for new growth. Plants in 400/0 μmol⋅m^−2^⋅s^–1^ treatment had a higher SLA than those in treatments with smaller PPFD fluctuations, possibly in an attempt to produce as much leaf as possible with the limited carbohydrate supply. Smaller leaf area and reduced leaf number in response to a fluctuation light levels (900–90 μmol⋅m^−2^⋅s^–1^ every 4 min, compared to a constant PPFD of 250 μmol⋅m^−2^⋅s^–1^) has also been reported in *Arabidopsis thaliana* (Kaiser et al., 2020).

### Fluctuating Light and Photosynthesis

Since plants in our study were exposed to fluctuating PPFD, their photosynthetic processes had to constantly respond to those changing conditions. Steady state A_n_ is typically achieved within 5–10 min of exposure to high PPFD (Kalaji et al., 2014). In our study, steady-state A_n_ was achieved within 2 min after exposure to a high PPFD in the 240/160, 280/120, and 320/80 μmol⋅m^−2^⋅s^–1^ treatments (Figure 4). This fast response to a change from low to high PPFD suggests that the photosynthetic apparatus in those plants was adequately activated under low PPFD to allow for a rapid response to an increase in PPFD. However, when PPFD was increased from 0 to 400 μmol⋅m^−2^⋅s^–1^ or from 40 to 360 μmol⋅m^−2^⋅s^–1^, A_n_ initially increased rapidly, followed by a more gradual increase during the remainder of the 15 min period, never reaching a steady state (Figure 4), suggesting that activation of the photosynthetic apparatus in response to a rapid change in PPFD depends on the magnitude of the change in PPFD. Sims and Pearcy (1993) grew the understory species *Alocasia macrorrhiza* with sunflecks for 10–12 min every hour (PPFD of ∼280 μmol⋅m^−2^⋅s^–1^ sunflecks alternating with ∼16 μmol⋅m^−2^⋅s^–1^ during the remainder of the hour) and without sunflecks. Plants in both treatments receiving a similar DLI. Induction of full photosynthetic activity in response to a sunfleck required ∼40 min, consistent with our observation that plants in the 400/0 and 360/40 μmol⋅m^−2^⋅s^–1^ treatments did not achieve steady-state photosynthesis during the 15 min at high PPFD. Exposing plants to sunflecks reduced leaf carbon gain, dry mass (by 89%) and increased SLA (Sims and Pearcy, 1993), similar to our findings in the 400/0 and 360/40 μmol⋅m^−2^⋅s^–1^ treatments.

Surprisingly, A_n_,_15_ decreased as PPFD increased from 320 to 400 μmol⋅m^−2^⋅s^–1^ (Figure 5). This indicates that large PPFD fluctuations negatively affect the photosynthetic performance of lettuce leaves. Leaf A_n_ depends on light harvesting, subsequent electron transport in the light reactions of photosynthesis, and the ability of Calvin cycle enzymes to use the products of the light reactions to assimilate CO_2_. Pigments in the thylakoid membrane of chloroplasts absorb light energy (photons) and that energy is used to drive electron transport. This results in the reduction of ferredoxin, followed by the reduction of NADP^+^ to NADPH (Pinnola, 2019) and the formation of a hydrogen gradient across the thylakoid membrane. This hydrogen gradient facilitates the synthesis of ATP. The rate of the light reactions depends on how much light is absorbed by photosynthetic pigments. The CCI was lower in the 400/0 and 360/40 μmol⋅m^−2^⋅s^–1^ treatments as compared to the treatments with smaller light fluctuations (Figure 6). A low CCI is associated with low leaf absorptance (Bauerle et al., 2004) and would thus be expected to result in low electron transport rates, which may result in low rates of NADPH and ATP production. This is supported by Wei et al. (2020), who reported that fluctuating light inhibits photosystem I and II activity through upregulation of non-photochemical quenching in rice (*Oryza sativa*). This resulted in decreased electron transport and lower ATP synthase activity. Fluctuating light also interfered with stacking of the thylakoid membrane. Thus fluctuating PPFDs can have a strong impact on the light reactions of photosynthesis.

The low chlorophyll levels in the treatments with large PPFD fluctuations may be due to light-dependent nature of chlorophyll biosynthesis. A key step in chlorophyll biosynthesis is the conversion of protochlorophyllide to chlorophyllide, the immediate precursor to chlorophyll *a* and chlorophyll *b*. This process that is both NADPH- and light-dependent (via the enzyme protochlorophyllide oxidoreductase, POR) (Reinbothe and Reinbothe, 1996). The activation of POR is unique in that its activation depends on the absorption of photons by its substrate protochlorophyllide. This induces a conformational change in the enzyme, activating it. Further complicating the effect of light on POR activity is that plants have multiple POR genes. In *Arabidopsis thaliana*, PORA is expressed in the dark and its expression is strongly inhibited in the light, through a phytochrome mediated process. PORB and PORC, on the other hand have low expression levels in the dark, and expression of PORC is upregulated in the light, through phytochrome-interacting factors (Gabruk and Mysliwa-Kurdziel, 2015). Thus, both the transcript levels and activity of POR are light-dependent and it seems plausible that production of chlorophyll cannot proceed normally when leaves are exposed to constant large light fluctuations, consistent with the low CCI in the 400/0 μmol⋅m^−2^⋅s^–1^ treatment (Figure 2). The idea that the low A_n_,_15_ at PPFDs of 360 and 400 μmol⋅m^−2^⋅s^–1^ was due at least in part due to poor light absorptance is supported by the positive correlation between CCI and A_n_,_30_ (Figure 6). The low CCI in the 400/0 μmol⋅m^−2^⋅s^–1^ treatment may also have been caused partly by leaf morphological effects. Plants in the 400/0 μmol⋅m^−2^⋅s^–1^ treatment had a high SLA, i.e., low biomass per unit leaf area. Since CCI is an indicator of the amount of chlorophyll per unit leaf area, a high SLA is likely associated a low CCI. We did indeed find strong negative correlations between SLA and both CCI and A_n_,_30_ (Figure 7), consistent with prior findings (Brodersen and Vogelmann, 2010).

Large fluctuations in PPFD may also affect Calvin cycle activity. The activation of key Calvin cycle enzymes and the biochemical reactions of the Calvin cycle themselves depend on products of the light reactions. Specifically, activation of fructose-1,6-bisphosphatase, sedoheptulose-1,7-bisphosphatase, glyceraldehyde-3-phosphate dehydrogenase, and phosphoribulokinase requires thioredoxin, produced from reduced ferredoxin, for the reduction of regulatory disulfides (Michelet et al., 2013). Low activity of these enzymes can limit photosynthesis by limiting the regeneration of ribulose 1,5-bisphosphate (RuBP). In addition, Rubisco activase is light-dependent, since it relies on a high stroma pH, which results from hydrogen transport from the stroma into the lumen in the light reactions. Rubisco activase activity depends on NADPH and thus on the light reactions (Kleczkowski, 1994). Rubsico is inactive in the dark because of the binding of metabolites to its active site and depends on Rubisco activase to remove those metabolites (Zhang and Portis, 1999).

Thus, light is not only required to drive the light reactions, but also controls the production and activity of chlorophyll and Calvin cycle enzymes. Although our data do not shed light on which enzymatic processes may have been affected by large PPFD fluctuations, it seems likely that such fluctuations interfere with the development of photosynthetic machinery and normal CO_2_ assimilation. The low A_n_,_15_ at PPFDs of 360 and 400 μmol⋅m^−2^⋅s^–1^ resulted in low A_n_,_30_ in the 360/40 and especially 400/0 μmol⋅m^−2^⋅s^–1^ treatments. That low A_n_ was likely partly responsible for the relatively poor growth in the 360/40 and 400/0 μmol⋅m^−2^⋅s^–1^ treatments, since A_n_,_30_ was strongly and positively correlated with shoot dry mass (Figure 6).

Stomatal conductance was also greatly affected by the light treatments (Supplementary Figure S2), with conductance decreasing with increasing PPFD fluctuations. Interestingly, conductance was not very responsive to the PPFD fluctuations themselves and remained stable during 15 min at high, followed by 15 min at low PPFD. The results may suggest that the low A_n_,_30_ in the 400/0 and 360/40 μmol⋅m^−2^⋅s^–1^ treatments may have been partly due to the low stomatal conductance in these treatments. However, that appears unlikely, given that the leaf internal CO_2_ concentration was 568 to 738 μmol⋅mol^–1^ and not affected by treatment. These relatively high leaf internal CO_2_ concentrations are unlikely to seriously limit CO_2_ assimilation. The differences in stomatal conductance thus seem to have been the result, rather than the cause, of the differences in A_n_,_30_.

### Practical Implications

‘Little Gem’ and ‘Green Salad Bowl’ lettuce tolerate fluctuating light levels, as long as the fluctuations are not extreme. This is consistent with the findings of Sørensen et al. (2016) and suggests that regulating supplemental light in response to real-time electricity prices is feasible for controlled environment agriculture. Our research was limited to two lettuce cultivars, which generally behaved similarly. Follow-up research on spreading (e.g., strawberry) and vine crops (e.g., tomato bell peppers, cucumbers) is needed to determine how other crops respond. In addition, we only tested fluctuations at 15 min intervals and how plants respond to different intervals is not clear. Although we did not answer all questions related to fluctuating lights, this research indicates that there is potential to reduce the electricity costs associated with supplemental lighting in response to real-time electricity price fluctuations. Dynamic algorithms that control supplemental lighting in response to variable sunlight conditions (Seginer et al., 2006) could be updated to incorporate real time pricing and implemented in the greenhouse industry. Such algorithms have been described (Clausen et al., 2015; Harbick et al., 2016; Sørensen et al., 2016), but it is not clear if they have been implemented in commercial greenhouses.

## Concluding Remarks

Our results indicate that lettuce can tolerate a wide range of fluctuating light levels. A constant PPFD is not needed to maintain proper growth and development of ‘Little Gem’ and ‘Green Salad Bowl.’ Extreme fluctuations, 400/0 μmol⋅m^−2^⋅s^–1^ and to a lesser extent the 360/40 μmol⋅m^−2^⋅s^–1^ treatments, resulted in plants with fewer and smaller leaves, lower chlorophyll content, and lower assimilation rates compared to those in all other treatments. However, results with smaller PPFD fluctuations indicate that growers can take advantage of variable electricity prices to provide light in controlled environment operations. This can aid growers in reducing operating costs and increase profitability.

## Data Availability Statement

The raw data supporting the conclusions of this article will be made available by the authors, without undue reservation.

## Author Contributions

RB and MI designed the experiment, discussed the data, and revised the manuscript. RB performed the experiment, analyzed the data, and prepared the first draft. Both authors contributed to the article and approved the submitted version.

## Conflict of Interest

The authors declare that the research was conducted in the absence of any commercial or financial relationships that could be construed as a potential conflict of interest.

## Supplementary Material

Supplementary Figure 1 Images of ‘Little Gem’(left and middle top) and ‘Green Salad Bowl’ (right and middle bottom) lettuce (*Lactuca sativa*) grown at PPFDs of 200/200 and 400/0 μmol⋅m^−2^⋅s^–1^ treatments, fluctuating at 15 min intervals.

Supplementary Figure 2 Stomatal conductance of‘Green Salad Bowl’ lettuce (*Lactuca sativa*) during a 15 min high PPFD period followed by a 15 min low *PPFD* period (∼400/0, 360/40, 320/80, 280/120, 240/160, and 200/200 μmol⋅m^−2^⋅s^–1^).
