# Supplementary material for: Only Extreme Fluctuations in Light Levels Reduce Lettuce Growth Under Sole Source Lighting
Source: Front Plant Sci. 2021 Jan 28;12:619973. doi: 10.3389/fpls.2021.619973 (PMC7875872; doi:10.3389/fpls.2021.619973)
Supplement: Supplementary Figure 1 — Images of ‘Little Gem’(left and middle top) and ‘Green Salad Bowl’ (right and middle bottom) lettuce (Lactuca sativa) grown at PPFDs of 200/200 and 400/0 μmol⋅m−2⋅s–1 treatments, fluctuating at 15 min intervals. [file Image_1.pdf]

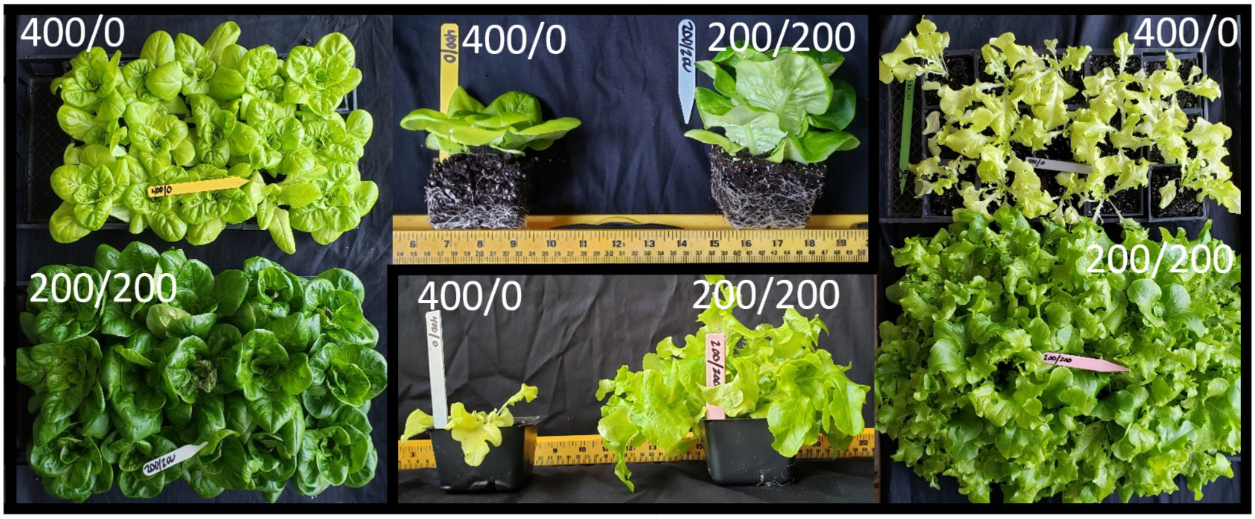

Supplementary figure 1. Images of ‘Little Gem’ (left and middle top) and ‘Green Salad Bowl’ (right and middle bottom) lettuce (*Lactuca sativa*) grown at *PPFD*s of 200/200 and 400/0 $\mu mol \cdot m^{-2} \cdot s^{-1}$ treatments, fluctuating at 15-minute intervals.

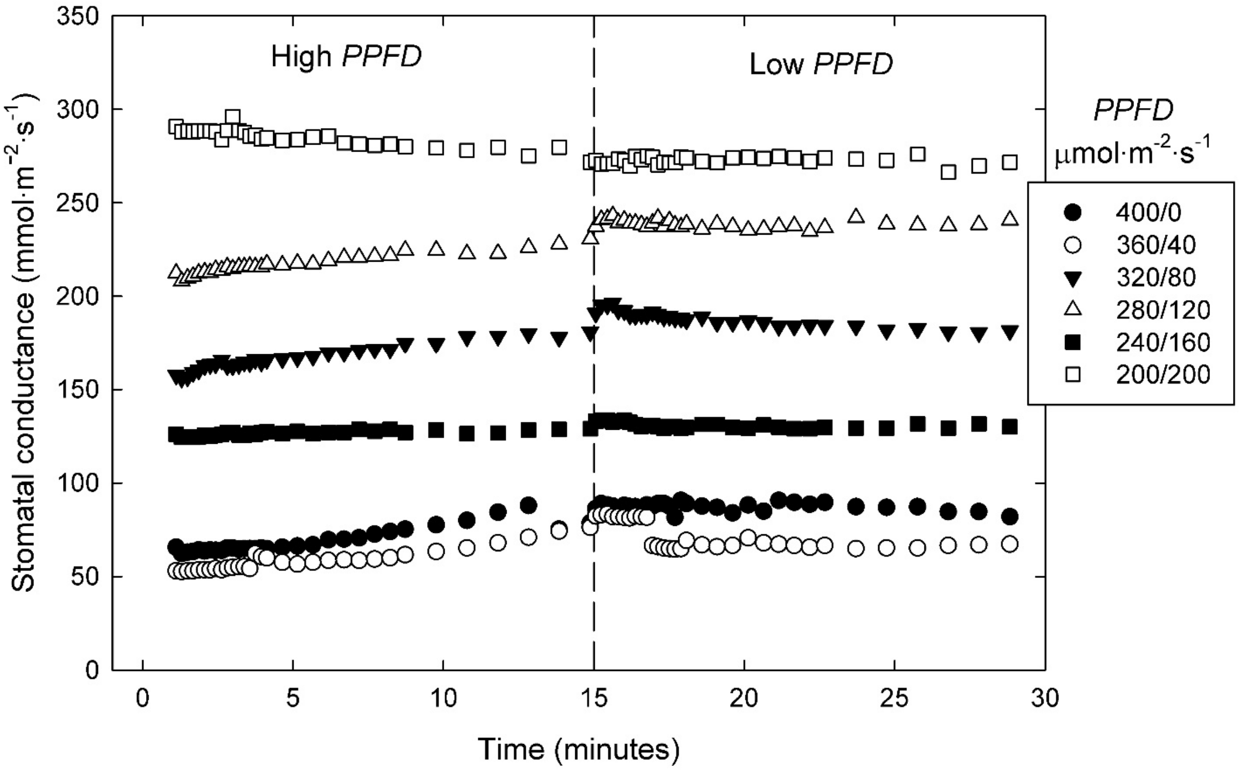

Supplementary figure 2. Stomatal conductance of 'Green Salad Bowl' lettuce (*Lactuca sativa*) during a 15-minute high photosynthetic photon flux density (*PPFD*) period followed by a 15-minute low *PPFD* period (~400/0, 360/40, 320/80, 280/120, 240/160, and 200/200 $\mu mol \cdot m^{-2} \cdot s^{-1}$).
